# Therapeutic efficacy and safety of S-1-based combination therapy compare with S-1 monotherapy following gemcitabine failure in pancreatic cancer: a meta-analysis

**DOI:** 10.1038/srep36944

**Published:** 2016-11-11

**Authors:** Sinan Lu, Yuan Zhang, Xiaohu Zhou, Dongkai Zhou, Qifan Yang, Bingjie Ju, Xinyi Zhao, Zhenhua Hu, Haiyang Xie, Lin Zhou, Shusen Zheng, Weilin Wang

**Affiliations:** 1 Key Lab of Combined Multi-Organ Transplantation, Ministry of Public Health, The First Affiliated Hospital, College of Medicine, Zhejiang University, Hangzhou 310003, China; 2 Division of Hepatobiliary and Pancreatic Surgery, Department of Surgery, First Affiliated Hospital, Zhejiang University School of Medicine, Hangzhou 310003, China; 3 Collaborative Innovation Center for Diagnosis and Treatment of Infectious Diseases, Hangzhou 310003, China

## Abstract

S-1 monotherapy is widely used following gemcitabine failure in pancreatic cancer, especially in East Asia. We performed a meta-analysis to determine whether S-1-based combination therapy had better efficacy and safety compared with S-1 monotherapy. We searched Pubmed, Web of Science, ClinicalTrials.gov, and Cochrane CENTRAL and subsequently included five trials with a total of 690 patients. The combined hazard ratio (HR) or risk ratio; the corresponding 95% confidence intervals of progression-free survival, overall survival, and overall response rate; and grade 3–4 adverse events were examined. Five randomized controlled trials were included. Meta-analysis demonstrated S-1-based combination therapy significantly increased progression-free survival (HR = 0.78, 95% confidence interval [CI]: 0.67–0.90, p = 0.0009) and overall response rate (HR = 1.74, 95% CI: 1.20–2.52, p = 0.003). Evidence was insufficient to confirm that S-1-based combined regimens improved overall survival (HR = 0.87, 95% CI: 0.75–1.00, p = 0.05). There was no significant difference in adverse events between the two treatment arms. In conclusion, S-1-based combination therapy improved progression-free survival and overall response rate compared to S-1 monotherapy with acceptable toxicity.

Pancreatic cancer has a low incidence but high mortality and is the 7^th^ leading cause of cancer-related mortality worldwide in both men and women^1^, although it is the 4^th^ leading cause in the United States^2^. Because of the low chance of successful surgery, chemotherapy is the most common approach to improve the survival time and life quality of the patients with pancreatic cancer.

One chemotherapeutic drug used for pancreatic cancer is S-1, which is an oral pro-drug of 5-fluorouracil (FU) and contains tegafur, gimeracil, and oteracil potassium in a 1.0:0.4:1.0 ratio^3^. Of these three components, tegafur is a pro-drug of 5-FU, which can be converted to 5-FU in the liver. Gimeracil maintains the concentration of 5-FU in serum and tumours by inhibiting dihydropyrimidine dehydrogenase, a vital enzyme which degrades 5-FU^4^. Oteracil, which inhibits phosphorylation of S-1, helps to reduce gastrointestinal side effects^5^. S-1 has been used in the treatment of several cancers, such as gastric cancer, colorectal cancer, and pancreatic cancer. In several phase II/III studies regarding gastric cancers^6^ or colorectal cancers^7^,^8^, S-1 was accepted because of similar efficacy and less complications compared with 5-FU infusion regimens.

For advanced pancreatic cancer, gemcitabine monotherapy is the standard first choice as a front-line chemotherapy. In a phase III randomized controlled trial (RCT), pancreatic cancer patients receiving gemcitabine showed a significant improvement of median overall survival (OS; 22.8 months vs 20.2 months) and 5-year survival rate (20.7% vs 10.4%) compared with the control group, respectively^9^. However, as use of gemcitabine in clinics has become more common for pancreatic cancer patients, refractoriness has emerged as a problem. Therefore, it is vital to find effective second-line therapies.

As reported previously, second-line chemotherapy may demonstrate better OS compared to the current best supportive care for gemcitabine-failed advanced pancreatic cancer^10^,^11^. In recent phase II RCTs, S-1 monotherapy demonstrated mildly improved efficacy with acceptable toxicity^12^,^13^. However, as the efficacy of S-1 monotherapy is not remarkable, different types of S-1-based combination therapies against advanced pancreatic cancer have been tested in several RCTs. For example, Mizuno *et al*.^14^ reported a phase II RCT comparing S-1 with S-1 plus irinotecan, and Ohkawa *et al*.^15^ published a phase II RCT comparing S-1 with S-1 plus oxaliplatin. However, in both cases, there was no difference in OS and progression-free survival (PFS) between the two treatment arms. More recently, a phase II RCT reported by Ueno *et al*.^16^ comparing S-1 with S-1 plus leucovorin demonstrated significantly improved PFS with the combination therapy (3.8 months vs 2.7 months, hazard ratio [HR] = 0.56, p = 0.003).

To determine the efficacy and safety of S-1-based combination regimens for pancreatic cancer, we conducted a meta-analysis with a subgroup analysis based on specific regimens or countries.

## Results

### Study inclusion and characteristics of included studies

**Figure 1:**
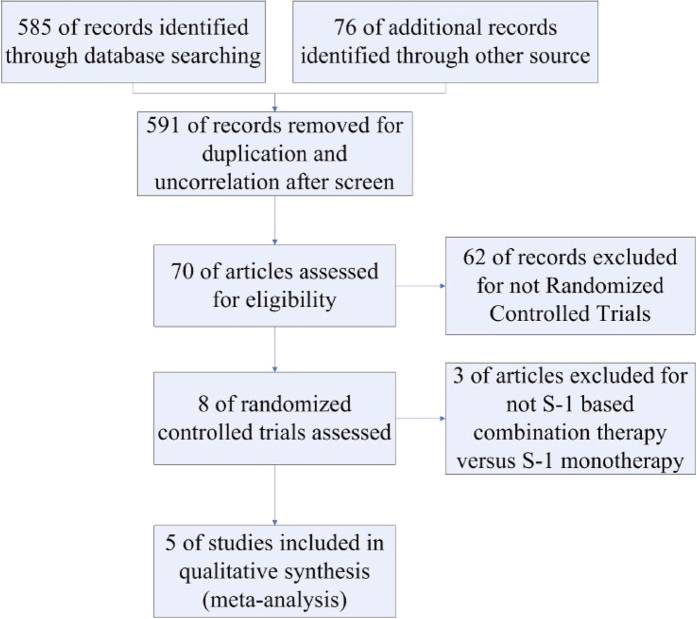
Flow program of the study enrollment process.

After strictly screening the articles based on the inclusion criteria, the number of articles was reduced from 585 to 5, including four full articles and one abstract. The search process is shown in Fig. 1. Using the five articles, a total of 690 patients were randomly distributed to either an S-1-based combination therapy group (340 patients) or S-1 monotherapy group (350 patients). Basic characteristics of the study, including chemotherapy regimens, are shown in Table 1.

Eligibility criteria of these five trials were accordant: (1) pancreatic carcinoma demonstrated by histological or cytological results, (2) failed gemcitabine-based chemotherapy, (3) no prior adjuvant radiotherapy, (4) Eastern Cooperative Oncology Group performance status score ≤2 (0–1, 96.5%; 2, 3.5%), and (5) age ≥18 years. Basic patient characteristics such as sex and primary tumor cite were showed in detail in Table 2. The Chi-square Test results of these characteristics were showed in Supplementary Fig. S1, All of the p value > 0.05, which means these characteristics between the two arms are well balanced.

### Quality assessment

Of the five trials included, two were from China, and three were from Japan. The four full articles described proper randomization, whereas the single abstract provided no details of patient allocation. None of the studies were blinded. Validity was assessed in detail (Supplementary Fig. S2), the score of the trials varies from 3–5.

### Efficacy

#### Primary end point: PFS

**Figure 2:**
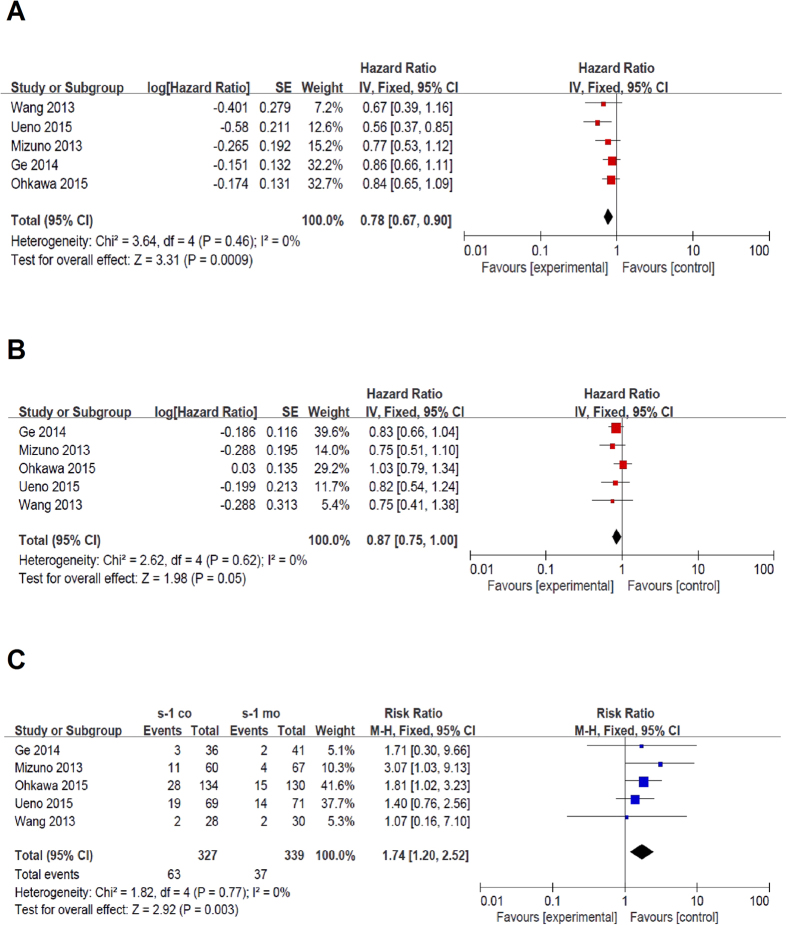
Standard forest plots of the major efficacy outcomes. (**A**) shows HR and 95% CI for PFS. (**B**) shows the HR and 95% CI for OS. (**C**) shows the risk ratio and 95% CI for ORR. S-1-based combination therapy is favorable when values lower than 1.

All five articles reported data regarding PFS. Median PFS was 3.3 months and 2.5 months in the S-1-based combination therapy and S-1 monotherapy groups, respectively. Pooled HR for PFS using a fixed-effects model was 0.78 (95% CI: 0.67–0.90, p = 0.0009), indicating improved PFS with S-1-based combination therapy. Furthermore, there was no proof of heterogeneity (p value of Q test = 0.46, I^2^ = 0%, Fig. 2A).

#### Secondary end points: OS and ORR

All five articles reported data regarding OS. Median OS was 6.9 months and 6.2 months in the S-1-based combination therapy and S-1 monotherapy groups, respectively. Pooled analysis in a fixed-effects model revealed borderline significance between groups (HR = 0.87, 95% CI: 0.75–1.00, p = 0.05, Fig. 2B). There was no heterogeneity in the above comparison (p value of Q test = 0.62, I^2^ = 0%).

All five articles reported data regarding ORR. Median ORR was significantly better in the S-1-based combination therapy group (19.2%) compared with that in the S-1 monotherapy group (10.8%) (HR = 1.74, 95% CI: 1.20–2.52, p = 0.003) in a fixed-effects model pooled analysis. No heterogeneity was indicated (p value of Q test = 0.77, I^2^ = 0%, Fig. 2C) between the trials.

### Safety

All five trials reported adverse events. Ten Grade 3 or higher side effects were divided into either hematologic or non-hematologic events. Haematologic events included leucopenia, neutropenia, thrombocytopenia, and anaemia. In addition to anaemia, the other three haematologic side effects occurred more frequently in the S-1-based combination therapy group. Non-haematological events included nausea/vomiting, diarrhoea, fatigue, anorexia, stomatitis, and elevation of bilirubin. Similar with the haematological events, all of the non-haematological side effects happened more commonly in the S-1-based combination therapy group. But all of the events were not significantly different between treatment groups (detailed in Table 3).

### Subgroup analysis

As the initial outcome of OS was borderline significant (p = 0.05), we further analysed OS in two subgroups: SL (S-1 plus leucovorin) and non-SL, depending on the regimen of S-1-based combination therapy, two trials from China and Japan were allocated into the SL group. No heterogeneity were demonstrated in SL/non-SL groups (p value of Q test = 0.06/0.35, I^2^ = 0%/10%, respectively). We also analysed based on the country of the study, either China (two trials including 150 patients) or Japan (three trials including 537 patients), neither subgroup analysis was significant as well. (Detailed in Supplementary Fig. S3).

### Publication bias

As the number of enrolled trials was only five, the analysis of publication bias was not that necessary actually. The shape of funnel plots showed no publication bias (Supplementary Fig. S4).

## Discussion

S-1, as a pro-drug of 5-FU, has been widely used in multiple levels of treatment for pancreatic cancer, especially in Asia. In the first multi-centric clinical study (GEST study) reported by Ueno *et al*.^17^, focused on locally advanced or metastatic pancreatic cancer, S-1 monotherapy was not inferior compared with gemcitabine monotherapy. In another Phase III study, S-1 monotherapy was superior as an adjuvant chemotherapy to gemcitabine after resection of pancreatic cancer^18^. However, there were not enough RCTs to directly compare S-1 and gemcitabine. Clinically, S-1 has always been used in combination with gemcitabine for unresectable pancreatic cancer. Depending on recent RCTs, several meta-analyses have shown gemcitabine plus S-1 significantly improves OS, PFS, ORR, and 1-year survival rate^19^,^20^,^21^,^22^.

In the current meta-analysis, we focused on the efficacy and safety of S-1 in gemcitabine-failed pancreatic cancer. We found that PFS and ORR in the S-1-based combination therapy group significant increased compared to those in the S-1 monotherapy group, with no significant difference in the adverse events between the two arms. This suggests that S-1-based combination therapy has a better response and disease control with acceptable toxicity compared with S-1 monotherapy in pancreatic cancer patients following gemcitabine failure.

OS also showed a 0.7-month increase in the S-1-based combination therapy group, although this was only of borderline significance. Subsequent subgroup analyses according to the regimen and country revealed no significant difference as well. Thus, despite some evidence that S-1-based combination therapy may increase OS, further evidence is still required.

Based on the above results, we speculate several potential reasons responsible for the insufficient evidence: (i) the number of trials was insufficient; (ii) the chemotherapy regimens used in S-1-based combination therapy groups were variable, with only S-1 plus leucovorin included in two articles; (iii) all five trials were from east Asia (China and Japan), thus the results need to be supplemented and confirmed with data from other geographic regions. (iv) The different post-protocol treatment: depending on the responses of patients after the using of chemotherapy, clinical doctors need to decide whether to continue the current regimen or not. And after the termination of regimen, different kinds of regimens will be used. 3 of 5 trials described the post-protocal treatments in the articles, Ueno *et al*. reported that 27 patients (39.1%) and 30 patients (42.3%) were treated with post-protocol regimens in the SL group and S-1 group, respectively. S-1 monotherapy was used in the SL group and gemcitabine plus S-1 therapy in the S-1 group. In the article reported by Ohkawa *et al*., 75/72 patients received the third-line therapies in S-1/SOX arm, and the contents were similar. Wang *et al*. also mentioned that the different third-line regimens were used (without detailed information). As the OS is heavily influenced by the post-protocol treatment, the pooled analysis result of OS is influenced as well. However, as the feature of clinical trial, the doctors can’t control the individualized treatment so strictly by the protocol of RCT. So we set PFS as the primary end point which is not affected by the post-protocol treatment.

Although OS between the two arms was not significantly different and influenced by the different post-protocol treatment, the apparent trend indicates that this is worth further study. Unfortunately, because of the lack of trials with the same regimen, we were unable to assess the efficacy of specific regimens. To overcome these limitations, additional RCTs are needed to increase the number of patients as well as to balance patients’ basic characteristics. This will enable determination of the efficacy between the different regimens using a network meta-analysis, which can provide information regarding the optimal regimen. Subsequent RCTs could then be designed to confirm the results.

In addition to the comparison of the S-1-based combination therapy and S-1 monotherapy groups, there is also a need to determine if there are better chemotherapy combinations not based on S-1 in the treatment of pancreatic cancer patients who have failed gemcitabine treatment. For instance, in a 2014 report of a phase III RCT comparing oxaliplatin, folinic acid, and fluorouracil to folinic acid and fluorouracil, Oettle *et al*.^23^ reported that the group receiving oxaliplatin showed significantly improved OS (HR = 0.66, 95% CI: 0.48–0.91, p = 0.010), PFS (HR = 0.68, 95% CI: 0.50–0.94, p = 0.019), and ORR (38.2% vs 7.1%, p < 0.001). Moreover, a double-blind phase II RCT with ruxolitinib plus capecitabine versus placebo plus capecitabine demonstrated no significant difference between the two treatment arms regarding OS and PFS^24^. Additionally, another phase II RCT showed no significant difference in OS between a group receiving selumetinib (5.4 months) and one receiving capecitabine (5.0 months)^25^. Other regimens, such as S-1 plus radiotherapy, S-1 with a fixed dose of gemcitabine, and paclitaxel plus S-1 have been used for pancreatic cancer following gemcitabine failure^26^,^27^,^28^, but RCTs have not been conducted for further verification.

In conclusion, after screening treatments for pancreatic cancer following gemcitabine failure, S-1 emerged as a potential option, especially in East Asia. The current meta-analysis demonstrated that S-1-based combination regimens significantly improved PFS and ORR, although effects on OS remain unclear and require further large RCTs from eastern and western countries for confirmation.

## Methods

### Search strategy and study selection

Two reviewers (S.L. and Y.Z.) independently searched Pubmed (1950–April 2016), Web of Science (1950–April 2016), ClinicalTrials.gov (to April 2016), and Cochrane CENTRAL (to April 2016). We used ‘pancreatic cancer’, ‘S-1’, and ‘gemcitabine’ as major terms of the search, The detailed search strategy of Pubmed was shown in Supplementary Fig. S5. We also searched abstracts published by the American Society of Clinical Oncology from 1990 to April 2016. The deadline for all studies was April 20, 2016. There were no language or geographical limitations. Inclusion criteria included (i) that it was a RCT for pancreatic cancer, (ii) that patients had failed first-line gemcitabine therapy, (iii) that an S-1-based combination therapy was compared to S-1 monotherapy, and (iv) that at least one of the following was reported: OS, PFS, or overall response rate (ORR). Any disagreements between the two reviewers were decided by consensus or by a third author (D.Z.).

### Data extraction and validity assessment

Detailed information extracted from the articles included (i) basic information, such as author, publication year, and type of publication; (ii) patient characteristics and treatment information; and (iii) efficacy and safety outcomes.

Risk of bias was determined using the Cochrane collaboration tool, including six items: (i) sequence generation, (ii) allocation concealment, (iii) blinding, (iv) incomplete outcome data, (v) no selective outcome reporting, and (vi) other sources of bias. For each item, a low risk counts as a score of 1, with a total of 6 scores. Two reviewers (B.J. and Q.Y.) independently assessed each category and reached a consensus by discussion or by including a third author (X.Z.).

### Statistical analysis

The primary end point was PFS, and OS and ORR were considered as secondary end points. PFS was defined as the time of trial randomization to disease progression or the last time available without progression. OS was defined as the time of trial randomization to death or the last time available. ORR as the sum of partial response rate and complete response rate, measured by Response Evaluation Criteria in Solid Tumours criteria. To determine the safety profile, Grade 3 or higher adverse events were extracted as per the Common Terminology Criteria for Adverse events.

Review Manager (Revman) version 5.3 (The Nordic Cochrane Center, The Cochrane Collaboration, Copenhagen, Denmark) was used to estimate HR, 95% confidence intervals (CI) of PFS and OS, risk ratio of ORR, and adverse events. If HRs and 95% CIs were not reported, they were estimated from other available data by using previously published methods^29^. Two-sided p values < 0.05 was defined as significant. A p value for Cochrane’s Q test of <0.1 or I^2^ > 50% was considered to show significant heterogeneity between the studies. Depending on the heterogeneity status, a random-effects model or fixed -effects model was utilized. Publication bias was estimated by funnel plots.

## Additional Information

**How to cite this article**: Lu, S. *et al*. Therapeutic efficacy and safety of S-1-based combination therapy compare with S-1 monotherapy following gemcitabine failure in pancreatic cancer: a meta-analysis. *Sci. Rep.* **6**, 36944; doi: 10.1038/srep36944 (2016).

**Publisher’s note:** Springer Nature remains neutral with regard to jurisdictional claims in published maps and institutional affiliations.

## Supplementary Material

Supplementary Information

## Figures and Tables

**Table 1:** Basic characteristics of the enrolled trials.

Ref.	Recruitment Duration	Country	Article Type	Study Design	Treatment						outcome measure	p Value
					No. of patients	Intervention		No. of patients	Control			
						Schedule of administration	Cycle Duration		Schedule of Administration	Cycle Duration		
Mizuno^14^	Nov 2008–Mar 2011	Japan	Abstract	RCT phase II	60	IRIS:CPT-11 100 mg/m2 iv, d1, 15 plus S-1 80/100/120 mg/day based on BSA, po, d1-14	4 weeks	67	S-1:80/100/120 mg/day based on BSA, po, d1-28	6 weeks	1. PFS2. OS3. ORR	1. >0.052. >0.053. <0.05
Wang^30^	April 2009–Mar 2012	China	Full text	RCT phase II	28	S-1 +CIK:human IFN-γ50 ng/ml, human IL-2 300 U/ml, human rIL-1α 100 U/ml iv, d12/14/16/18/20 plus S-1 80/100/120 mg/day based on BSA, po, d1-21	4 weeks	30	S-1:80/100/120 mg/day based on BSA, po, d1-21	4 weeks	1. PFS2. OS3. DCR	1. <0.052. >0.053. >0.05
Ge^31^	Feb 2010–Oct 2013	China	Full text	RCT phase II	45	SL:leucovorin 25 mg bidpo, d1-14 plus S-1 80/100/120 mg/day based on BSA, po, d1-14	3 weeks	47	S-1:80/100/120 mg/day based on BSA, po, d1-14	3 weeks	1. PFS2. OS3. ORR	1. >0.052. >0.053. >0.05
Ueno^16^ 2015	Aug 2011–Aug 2012	Japan	Full text	RCT phase II	71	SL:leucovorin 25 mg bidpo, d1-7 plus S-1 80/100/120 mg/daybased on BSA, po, d1-28	2 weeks	71	S-1:80/100/120 mg/day based on BSA, po, d1-28	6 weeks	1. PFS2. OS3. DCR	1. <0.052. >0.053. <0.05
Ohkawa^15^	Jan 2009–July 2010	Japan	Full text	RCT phase II	136	SOX:oxaliplatin 100 mg/m2 iv, d1 plus S-1 80/100/120 mg/day based on BSA, po, d1-14	3 weeks	135	S-1:80/100/120 mg/day based on BSA, po, d1-28	6 weeks	1. PFS2. OS3. ORR	1. >0.052. >0.053. <0.05

**Table 2:** Basic characteristics of the patients.

characteristics	Wang^30^		Ge^31^		Ueno^16^		Ohkawa^15^	
	S-1 Co (n = 28)	S-1 Mo (n = 30)	S-1 Co (n = 45)	S-1 Mo (n = 47)	S-1 Co (n = 69)	S-1 Mo (n = 71)	S-1 Co (n = 134)	S-1 Mo (n = 130)
Age, yr								
Median	62	48	57	58	65	64	65	63.5
Rang	40–76	40–65	36–76	30–76	NA	NA	27–83	43–80
Others, No. (%)								
Male	15(53.6)	16(53.3)	26(57.8)	35(74.5)	41(59.4)	38(53.5)	82(61.2)	80(61.5)
ECOG score								
0	7(25.0)	8(26.7)	NA	NA	45(65.2)	48(67.6)	93(69.4)	92(70.8)
1	10(35.7)	9(30.0)	NA	NA	24(34.8)	23(32.4)	41(30.6)	38(29.2)
2	11(39.3)	13(43.3)	0(0)	0(0)	0(0)	0(0)	0(0)	0(0)
BSA, m2								
<1.25	NA	NA	NA	NA	3(4.3)	2(2.8)	8(6.0)	6(4.6)
1.25–1.5	NA	NA	NA	NA	28(40.6)	28(39.4)	53(39.6)	51(39.2)
>1.5	NA	NA	NA	NA	38(55.1)	41(57.8)	73(54.4)	73(56.2)
Primary tumor site								
head	22(78.6)	23(76.7)	19(42.2)	21(44.7)	NA	NA	38(28.4)	34(26.2)
others	6(22.4)	7(23.3)	26(57.8)	26(55.3)	NA	NA	106(71.6)	106(73.8)
pancreatectomy								
Yes	NA	NA	21(46.7)	21(44.7)	13(18.8)	24(33.8)	NA	NA
No	NA	NA	24(53.3)	26(55.3)	56(81.2)	47(66.2)	NA	NA

**Table 3:** Analytic results of adverse events.

Adverse Events, %	Mizuno *et al*.^14^		Wang *et al*.^30^		Ge *et al*.^31^		Ueno *et al*.^16^		Ohkawa *et al*.^15^		Total		
	S-1 based(n = 60)	S-1 mono(n = 67)	S-1 based(n = 28)	S-1 mono(n = 30)	S-1 based(n = 45)	S-1 mono(n = 47)	S-1 based(n = 71)	S-1 mono(n = 71)	S-1 based(n = 136)	S-1 mono(n = 132)	S-1 based(n = 340)	S-1 mono(n = 347)	p Value
hematologic													
leukopenia	NA	NA	3.6	6.7	NA	NA	7.0	4.2	4.4	2.3	3.5	2.3	0.37
Neutropenia	15.6	4.3	3.6	3.3	4.4	4.3	8.5	5.6	8.1	11.4	8.5	7.2	0.63
Thrombocytopenia	NA	NA	0.0	0.0	0.0	2.1	2.8	0.0	10.3	4.5	4.7	2.0	0.08
Anemia	NA	NA	NA	NA	2.2	2.1	9.9	11.7	8.1	13.6	5.6	7.8	0.19
Non-hematologic													
Nausea/Vomiting	6.3	2.9	0.0	3.3	0.0	0.0	1.4	2.8	6.6	3.0	4.1	2.6	0.28
Diarrhea	3.1	2.9	7.1	6.7	13.3	2.1	5.6	4.2	5.1	6.1	6.2	4.6	0.37
Fatigue	NA	NA	0.0	3.3	6.7	4.3	7.0	0.0	2.9	3.8	2.4	2.3	0.38
Anorexia	23.4	17.3	NA	NA	NA	NA	NA	NA	14.7	12.9	10.0	8.4	0.42
Stomatitis	NA	NA	7.1	3.3	13.3	0.0	2.8	0.0	1.5	2.3	3.5	1.2	0.06
Bilirubin	NA	NA	0.0	0.0	2.2	4.3	NA	NA	11.8	4.5	5.0	3.5	0.37
