# Supplementary material for: Therapeutic efficacy and safety of S-1-based combination therapy compare with S-1 monotherapy following gemcitabine failure in pancreatic cancer: a meta-analysis
Source: Sci Rep. 2016 Nov 11;6:36944. doi: 10.1038/srep36944 (PMC5105146; doi:10.1038/srep36944)
Supplement: Supplementary Information [file srep36944-s1.pdf]

**Therapeutic efficacy and safety of S-1-based combination therapy compare with S-1 monotherapy following gemcitabine failure in pancreatic cancer: a meta-analysis**

Sinan Lu, [1+], Yuan Zhang, [1+], Xiaohu Zhou, [1], Dongkai Zhou, [1], Qifan Yang, [1] Bingjie Ju, [1], Xinyi Zhao, [1], Zhenhua Hu, [2], Haiyang Xie, [1], Lin Zhou, [1], Shusen Zheng, [2,3] * , Weilin Wang, [2,3] *

## Figure legends

Supplementary figure S1. Chi-Square Test results of patient characteristics. S1a shows the Chi-Square result of gender (male or female) between the two arms. S1b shows the Chi-Square result of ECOG score (0 or 1 plus 2) between the two arms. S1c shows the pancreatectomy status (yes or no) between the two arms. S1d shows the primary tumor cite (head or others) between the two arms. All of the p value > 0.05, which means the characteristics between the two arms are well balanced.

Supplementary figure S2. Validity assessments of articles by the Cochrane collaboration tool.

Supplementary figure S3. Standard forest plots of the subgroup analysis. S2a shows the subgroup analysis depending on the regimens of S-1 based combination therapy, SL means S-1 plus leucovorin. S2b shows the subgroup analysis depending on the country.

Supplementary figure S4. Funnel plots of the meta-analysis. S3a shows the funnel plot of PFS. S3b shows the funnel plot of OS. S3c shows the funnel plot of ORR. The shape of all funnel plots indicate no publication bias here.

Supplementary figure S5. Search strategy of Pubmed.

## Figures

1.Supplementary figure S1

a

Chi-Square Tests

| | Value | df | Asymp Sig. (2-sided) | Exact Sig.(2-sided) | Exact Sig.(1-sided) |
|---|---|---|---|---|---|
| Pearson Chi-Square | .109[a] | 1 | .742 | | |
| Continuity Correction[b] | .059 | 1 | .808 | | |
| Likelihood Ratio | .109 | 1 | .742 | | |
| Fisher's Exact Test | | | | .795 | .404 |
| Linear-by-Linear Association | .108 | 1 | .742 | | |
| N of Valid Cases | 554 | | | | |

b

Chi-Square Tests

| | value | df | Asymp Sig. (2-sided) | Exact Sig.(2-sided) | Exact Sig.(1-sided) |
|---|---|---|---|---|---|
| Pearson Chi-Square | .084[a] | 1 | .772 | | |
| Continuity Correction[b] | .037 | 1 | .847 | | |
| Likelihood Ratio | .084 | 1 | .772 | | |
| Fisher's Exact Test | | | | .847 | .423 |
| Linear-by-Linear Association | .084 | 1 | .772 | | |
| N of Valid Cases | 462 | | | | |

c

Chi-Square Tests

| | Value | df | Asymp Sig. (2-sided) | Exact Sig.(2-sided) | Exact Sig.(1-sided) |
|---|---|---|---|---|---|
| Pearson Chi-Square | 1.783[a] | 1 | .182 | | |
| Continuity Correction[b] | 1.433 | 1 | .231 | | |
| Likelihood Ratio | 1.788 | 1 | .181 | | |
| Fisher's Exact Test | | | | .213 | .116 |
| Linear-by-Linear Association | 1.776 | 1 | .183 | | |
| N of Valid Cases | 232 | | | | |

d

Chi-Square Tests

| | Value | df | Asymp Sig. (2-sided) | Exact Sig.(2-sided) | Exact Sig.(1-sided) |
|---|---|---|---|---|---|
| Pearson Chi-Square | .010[a] | 1 | .920 | | |
| Continuity Correction[b] | .000 | 1 | 1.000 | | |
| Likelihood Ratio | .010 | 1 | .920 | | |
| Fisher's Exact Test | | | | 1.000 | .500 |
| Linear-by-Linear Association | .010 | 1 | .921 | | |
| N of Valid Cases | 434 | | | | |

2.Supplementary figure S2

a

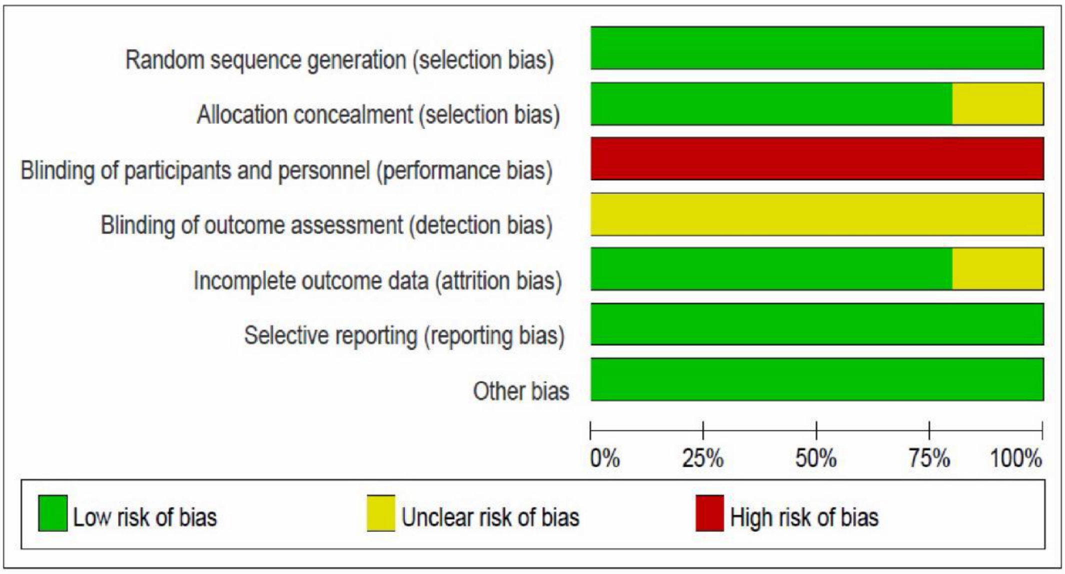


b

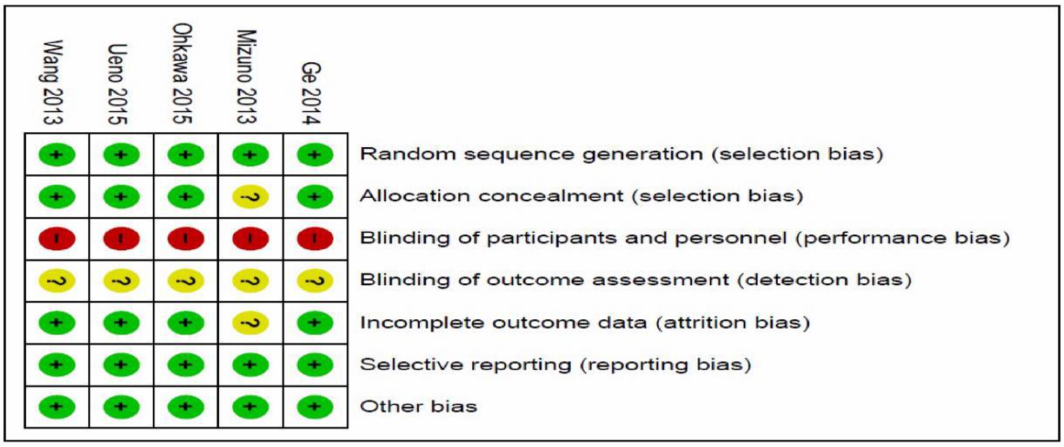


3.Supplementary figure S3

**a**

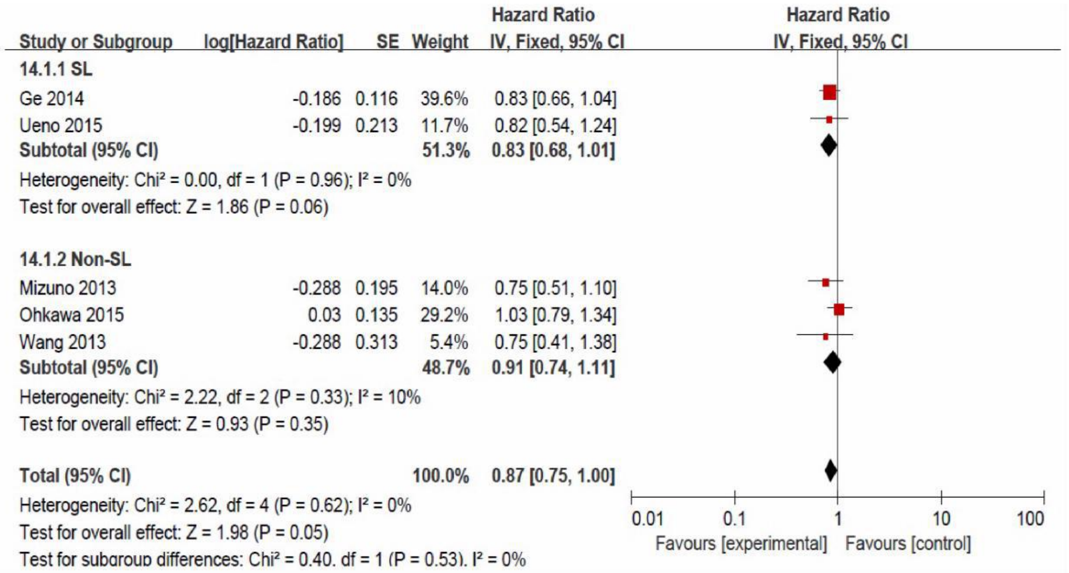

Study or Subgroup
log[Hazard Ratio]
SE
Weight
Hazard Ratio IV, Fixed, 95% CI
Hazard Ratio IV, Fixed, 95% CI
14.1.1 SL
Ge 2014 -0.186 0.116 39.6% 0.83 [0.66, 1.04]
Ueno 2015 -0.199 0.213 11.7% 0.82 [0.54, 1.24]
Subtotal (95% CI) 51.3% 0.83 [0.68, 1.01]
Heterogeneity: Chi² = 0.00, df = 1 (P = 0.96); I² = 0%
Test for overall effect: Z = 1.86 (P = 0.06)
14.1.2 Non-SL
Mizuno 2013 -0.288 0.195 14.0% 0.75 [0.51, 1.10]
Ohkawa 2015 0.03 0.135 29.2% 1.03 [0.79, 1.34]
Wang 2013 -0.288 0.313 5.4% 0.75 [0.41, 1.38]
Subtotal (95% CI) 48.7% 0.91 [0.74, 1.11]
Heterogeneity: Chi² = 2.22, df = 2 (P = 0.33); I² = 10%
Test for overall effect: Z = 0.93 (P = 0.35)
Total (95% CI) 100.0% 0.87 [0.75, 1.00]
Heterogeneity: Chi² = 2.62, df = 4 (P = 0.62); I² = 0%
Test for overall effect: Z = 1.98 (P = 0.05)
Test for subgroup differences: Chi² = 0.40, df = 1 (P = 0.53), I² = 0%
0.01
0.1
1
10
100
Favours [experimental]
Favours [control]


**b**

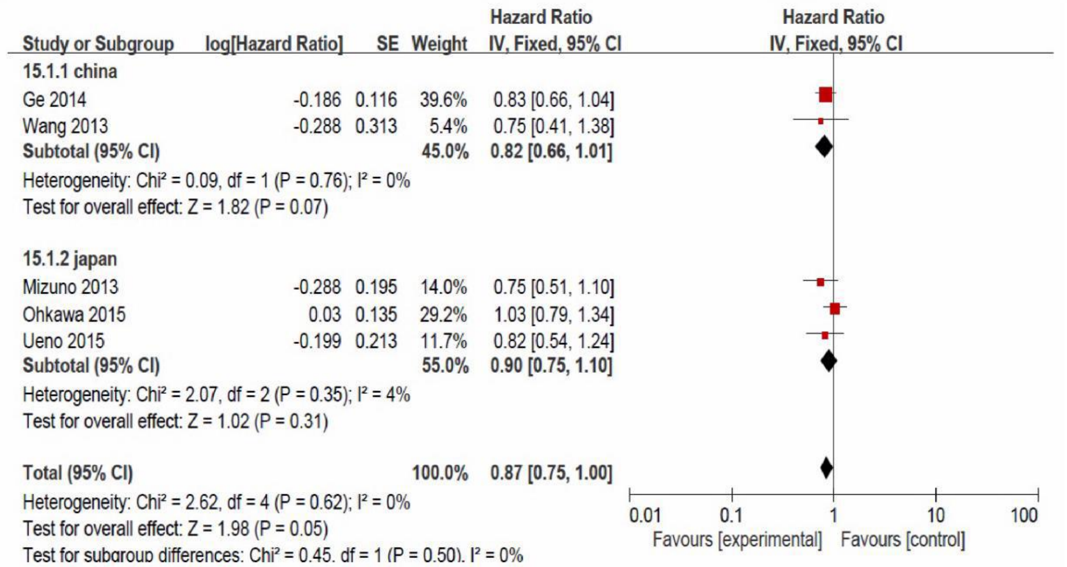

Study or Subgroup
log[Hazard Ratio]
SE
Weight
Hazard Ratio IV, Fixed, 95% CI
Hazard Ratio IV, Fixed, 95% CI
15.1.1 china
Ge 2014 -0.186 0.116 39.6% 0.83 [0.66, 1.04]
Wang 2013 -0.288 0.313 5.4% 0.75 [0.41, 1.38]
Subtotal (95% CI) 45.0% 0.82 [0.66, 1.01]
Heterogeneity: Chi² = 0.09, df = 1 (P = 0.76); I² = 0%
Test for overall effect: Z = 1.82 (P = 0.07)
15.1.2 japan
Mizuno 2013 -0.288 0.195 14.0% 0.75 [0.51, 1.10]
Ohkawa 2015 0.03 0.135 29.2% 1.03 [0.79, 1.34]
Ueno 2015 -0.199 0.213 11.7% 0.82 [0.54, 1.24]
Subtotal (95% CI) 55.0% 0.90 [0.75, 1.10]
Heterogeneity: Chi² = 2.07, df = 2 (P = 0.35); I² = 4%
Test for overall effect: Z = 1.02 (P = 0.31)
Total (95% CI) 100.0% 0.87 [0.75, 1.00]
Heterogeneity: Chi² = 2.62, df = 4 (P = 0.62); I² = 0%
Test for overall effect: Z = 1.98 (P = 0.05)
Test for subgroup differences: Chi² = 0.45, df = 1 (P = 0.50), I² = 0%
0.01
0.1
1
10
100
Favours [experimental]
Favours [control]

4.Supplementary figure S4

a

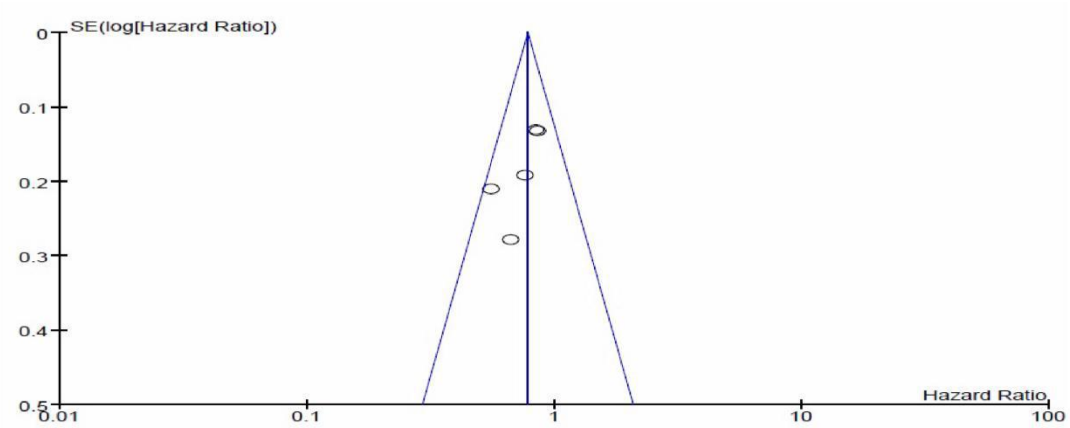


b

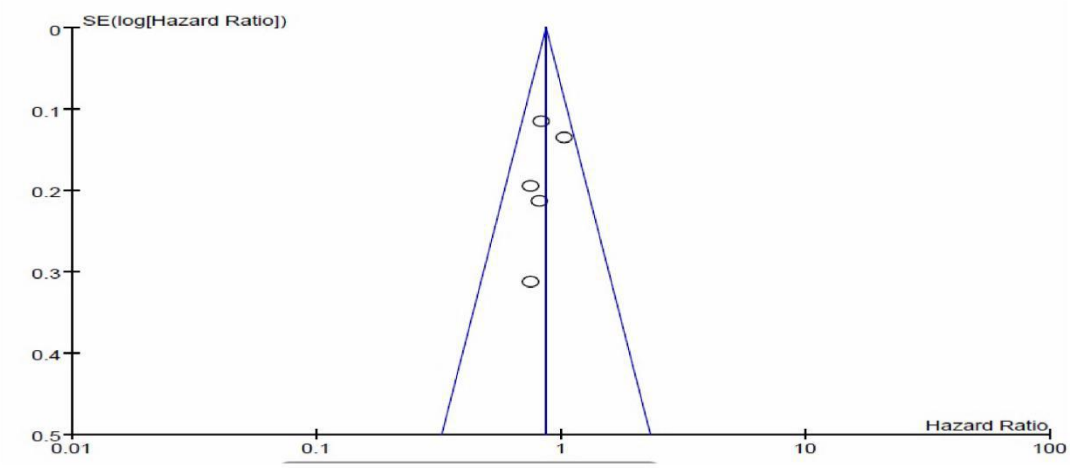


c

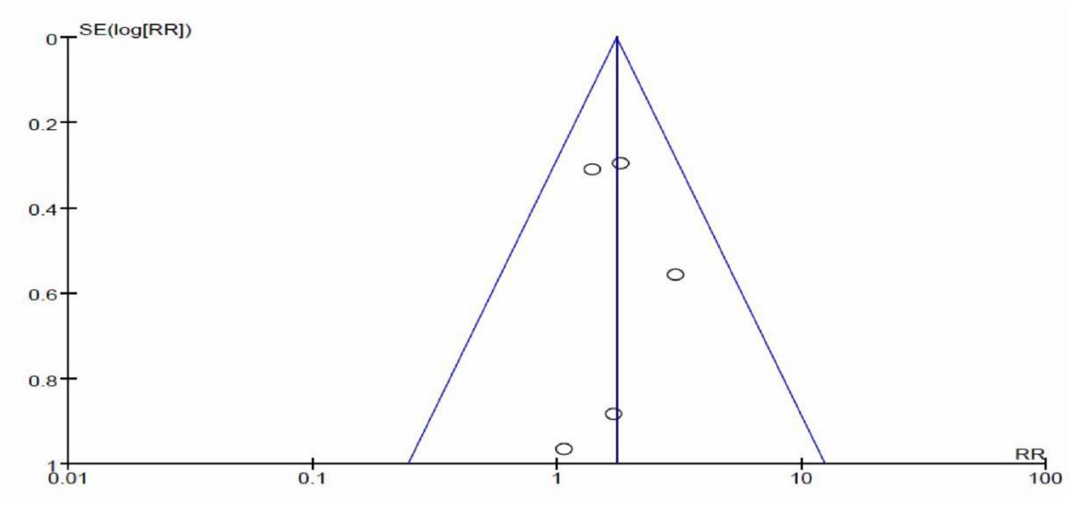

5.Supplementary figure S5

1. Pancrea*
2. S-1
3. 5-FU
4. Fluorouracil
5. OR/2-4
6. 1 AND 5
7. Gemcitabine
8. Refractory
9. Resist*
10. Fail*
11. OR/8-10
12. 7 AND 11
13. 6 AND 12[Title/Abstract]
14. 13 Filter:Review
15. 13 Filter:Case Report
16. 13 Filter:Comments
17. 13 Filter:Editorial
18. 13 Filter:Letter
19. 13 Filter:Other Animals
20. OR/14-19
21. 13 NOT 20
